# How and When Do Insects Rely on Endogenous Protein and Lipid Resources during Lethal Bouts of Starvation? A New Application for ^13^C-Breath testing

**DOI:** 10.1371/journal.pone.0140053

**Published:** 2015-10-14

**Authors:** Marshall D. McCue, R. Marena Guzman, Celeste A. Passement, Goggy Davidowitz

**Affiliations:** 1 St. Mary’s University, Department of Biological Sciences, San Antonio, Texas, United States of America; 2 University of Arizona, Department of Entomology, Tucson, Arizona, United States of America; University of Cincinnati, UNITED STATES

## Abstract

Most of our understanding about the physiology of fasting and starvation comes from studies of vertebrates; however, for ethical reasons, studies that monitor vertebrates through the lethal endpoint are scant. Insects are convenient models to characterize the comparative strategies used to cope with starvation because they have diverse life histories and have evolved under the omnipresent challenge of food limitation. Moreover, we can study the physiology of starvation through its natural endpoint. In this study we raised populations of five species of insects (adult grasshoppers, crickets, cockroaches, and larval beetles and moths) on diets labeled with either ^13^C-palmitic acid or ^13^C-leucine to isotopically enrich the lipids or the proteins in their bodies, respectively. The insects were allowed to become postabsorptive and then starved. We periodically measured the δ^13^C of the exhaled breath to characterize how each species adjusted their reliance on endogenous lipids and proteins as energy sources. We found that starving insects employ a wide range of strategies for regulating lipid and protein oxidation. All of the insects except for the beetle larvae were capable of sharply reducing reliance on protein oxidation; however, this protein sparing strategy was usually unsustainable during the entire starvation period. All insects increased their reliance on lipid oxidation, but while some species (grasshoppers, cockroaches, and beetle larvae) were still relying extensively on lipids at the time of death, other species (crickets and moth larvae) allowed rates of lipid oxidation to return to prestarvation levels. Although lipids and proteins are critical metabolic fuels for both vertebrates and insects, insects apparently exhibit a much wider range of strategies for rationing these limited resources during starvation.

## Introduction

All animals face the possibility of food limitation during which they must rely solely on endogenous nutrients to fuel their continued metabolic demands. Most of our understanding about how animals respond to starvation comes from studies of vertebrates (see reviews by [1–6]). While vertebrates demonstrate a number of physiological strategies for surviving starvation these strategies may not necessarily be generalizable to invertebrates such as insects that have also evolved under the omnipresent challenges of food limitation (e.g., [7–11]).

Insects outnumber vertebrates in number, species, and biomass [12]. Because they play central roles in most terrestrial and aquatic food webs [13] it is important to understand how these animals cope with fluctuating food resources. Although the diversity of starvation tolerance among insects may be no less impressive than that documented among vertebrate animals, we know quite little about how they physiologically respond to starvation [14–18]; in fact, recent reviews have explicitly cited the need for additional comparative studies of starvation among insects and other invertebrates [19–21].

### Characterizing the progression of starvation

Can researchers use the same physiological toolbox developed for vertebrate animals to study starvation in insects? Starvation-induced changes in blood metabolites used routinely in vertebrates (e.g., glucose, ketone bodies, and nitrogen metabolites) are rarely reported for insects in part because of logistical issues related to body size and peculiarities of hemolymph metabolites (e.g., trehalose and proline [18,22]), and perhaps also in response to the growing awareness that circulating metabolites offer limited mechanistic insight into systemic nutrient fluxes [3,23,24]. Indeed, studies have shown that starving insects mobilize glycogen and triglycerides stored in their fat bodies [17,18,22,25,26], but destructive sampling of body composition precludes continual measurements of fuel oxidation over long periods.

Changes in respiratory exchange ratios (RERs) of starving insects are often difficult to quantitatively interpret [15,19,27] and therefore may preclude accurate assessments of changes in metabolic fuel mixtures [28–30]. This limitation is confounded with the fact that VCO_2_ can usually be measured in insects with greater accuracy than VO_2_ [31]. Other non-invasive approaches like NMR microscopy can be used to quantify changes in the fat and water content in small insects over time and therefore may be used to estimate lipid oxidation [32]; but the method is not suitable for tracking changes in protein use or for making measurements in large numbers of individuals. Quantitative magnetic resonance (QMR) has been shown to be suitable for accurate measures of lean mass in large insects [33], but it is not suitable for quantifying the lipid content in their bodies.

Recently developed approaches where different nutrient pools in the body (e.g., carbohydrates, lipids, and proteins) are selectively enriched with a stable isotope (e.g., ^13^C) have been coupled with ^13^C-breath testing to characterize the starvation-induced changes in metabolic fuels among birds and mammals [34,35], but they have not yet been used to study starvation in insects. Here we examine whether insects of different species and age classes exhibit strategies of rationing oxidative fuels that are generally similar to those seen among most vertebrates. Based on the traditional three-phase paradigm about fuel switching (reviewed in [1,36–38]), it is likely that carbohydrates in the hemolymph provide a readily available fuel source and therefore lipid and protein oxidation are minimal at the onset of starvation. As starvation progresses, lipids are expected to become the predominant source of metabolic energy. Protein oxidation is expected to increase dramatically, but only during the lattermost phases of starvation when most, or all, of the lipid reserves have been depleted. Because of the ethical concerns of starving vertebrate animals to death, this pre-mortem increase in protein oxidation, often used to delimit the transition from phase II to phase III, is rarely documented in vertebrates. However, insects are convenient models in which to test the prediction that death from starvation is invariably preceded by a dramatic, unsustainable increase in protein oxidation (*sensu* [39]).

### Species selection

In contrast to vertebrates in which much of the work on fuel use during starvation has been done, insects have a greater diversity of life history strategies for growth and development. Insects of some orders are hemimetabolous, whereas others are holometabolous. In general, juveniles of hemimetabolous insects eat the same food as do the adults, whereas juveniles of holometabolous insects typically live in very different habitats and eat substantially different foods than do the adults. Some insects are capital breeders in which (nearly) all of the resources used for reproduction are acquired during the juvenile (larval) phase of growth [40,41]. In contrast, income breeders accumulate resources used in reproduction during the adult stage. Insects vary dramatically in size and insect growth is exponential [42] so that nearly all of growth occurs in the last larval instar [43]. Last, longevity of the juvenile stage relative to the adult stage and longevity of lifespan in general is extremely variable among insect species.

To account for this complexity of life histories we chose five species of insects that span a range of life history strategies with the restriction that we could rear sufficient numbers to accommodate the study. The Madagascar hissing cockroach, *Gromphadorhina portentosa* is hemimetabolous, large sized and relatively longed lived. The eastern lubber grasshopper, *Romalea microptera* is also hemimetabolous and large sized, but has a shorter lifespan. The house cricket, *Acheta domesticus*, is hemimetabolous but smaller with a shorter lifespan than the grasshoppers. The darkling beetle, *Zophobis morio* is a small holometabolous income breeder. The hawk moth, *Manduca sexta*, is a large holometabolous capital breeder. It was not our intention to exhaustively sample all insect life histories, rather, the insects used in this study represent a limited subset of insect life history strategies that represent a significant diversity of strategies to provide a picture of possible strategies of fuel use during starvation. As mentioned above, there are no studies of starvation strategies in insects. With this perspective as a starting point, subsequent studies can test specific hypotheses of starvation strategies across taxa, life history stages, and environmental conditions.

## Methods

### Animals and experimental diets

The five phylogenetically diverse species of insects were raised in the laboratory on one of two ^13^C-labeled tracers (^13^C-1-palmitic acid or ^13^C-1-L-leucine; Cambridge Isotope Laboratories, Tewksbury, MA) with the aim of isotopically enriching either their body lipids or proteins, respectively. They were then starved while we measured the δ^13^C in their exhaled breath to track how starvation affected their reliance on endogenous lipid and protein oxidation.

Madagascar hissing cockroaches (*G*. *portentosa*) nymphs (n = 300; age 1–14 days; 10–20mg) were selected from a larger colony maintained for several generations in our laboratory and randomly assigned to one of two diet treatment groups (Table 1). They were raised to adulthood (4–5 months of age) on a base diet consisting of ground chick starter food (Nutrena; Naturewise) supplemented with one of the two ^13^C-labeled tracers. Lots of n = 20 adult cockroaches from each population were placed into five metabolic chambers (1.5 L) where they were starved. The bottom of each metabolic chamber consisted of a false floor of 1cm˟1cm wire mesh that allowed feces to pass through thereby preventing coprophagy. Segments of PVC tubing were also placed inside the metabolic chambers to provide a hiding place where they naturally reside during the day. The cockroaches were given 24 hours to become postabsorptive before beginning the breath collection.

**Table 1:** Summary of the five insect species used and their experimental diets used to isotopically enrich their tissues.

Species	Base diet	^13^C-1-L-leucine	^13^C-1-palmitic acid
*Gromphadorhina portentosa*	Chick food	1 g kg^-1^	1 g kg^-1^
*Acheta domesticus*	Tilapia chow	1.667g kg^-1^	1 g kg^-1^
*Romalea microptera*	Romaine lettuce	variable	variable
*Manduca sexta*	Prepared diet	8 g kg^-1^	8 g kg^-1^
*Zophobis morio*	Bran+Tilapia chow	1 g kg^-1^	1 g kg^-1^

Bran was mixed in with the ^13^C-labeled tilapia chow for the larval beetles.

House crickets (*A*. *domesticus*) nymphs (n = 500; age 2 weeks) were obtained from a commercial vendor (Fluker Farms; Port Allen, LA) and were randomly divided into one of two diet treatment groups (Table 1). They were raised to adulthood (6-weeks of age) on a base diet of ground tilapia pellets mixed with one of the two ^13^C-labeled tracers. Lots of n = 40 adult crickets from each population were relocated into five metabolic chambers (1.0 L) where they were starved. The metabolic chambers were lined with 1cm˟1cm plastic mesh to provide three-dimensional contour. The crickets were given 12 hours to become postabsorptive before beginning the breath collection.

Eastern lubber grasshoppers (*R*. *microptera*) nymphs (n = 80, male, 2–3cm) were collected by Prof. John Hatle with permission of a private property owner in Jacksonville, Florida; no collecting permit was required. They were randomly assigned to one of two diet treatment groups and fed a base diet of romaine lettuce leaves lightly dusted with one of the two ^13^C-labeled tracers (Table 1). They were raised to adulthood over the following 6 weeks. Individual grasshoppers were placed inside 100ml plastic syringes that served as metabolic chambers. The grasshoppers were given 24 hours to become postabsorptive before beginning the breath collection.

Darkling beetles (*Z*. *morio*) larvae (mealworms; n = 100, <1cm) were selected from a colony maintained for several generations in our laboratory and randomly assigned to one of two diet treatment groups (Table 1). They were raised to their penultimate larval instar (~300–500mg) on a diet of unlabeled wheat bran and ground tilapia pellets mixed with one of the two ^13^C-labeled tracers (Table 1). Individual larvae were placed inside 20ml syringes that served as metabolic chambers. The beetle larvae were given 12 hours to become postabsorptive before beginning the breath collection.

Hawk moth (*M*. *sexta*) larvae (tobacco hornworms; n = 60) were hatched from eggs of adults from a colony maintained for several generations in our laboratory. They were raised to their penultimate larval instar (~300–500mg) on a prepared diet [44] supplemented with one of the two ^13^C-labeled tracers (Table 1). Individual larvae were placed inside 60ml syringes that served as metabolic chambers. The larvae were given 12 hours to become postabsorptive before beginning the breath collection.

For all species, ambient temperature was 28°C and photoperiod was 14L10D during the rearing and starvation trials. Breath samples were collected once or twice a day depending on the species. For group-housed animals (i.e., crickets and cockroaches) dead individuals or those who lost normal locomotory ability were removed from cages twice each day to prevent cannibalism. Determination of the post absorptive period was based on size with the three smaller species (cricket, beetle, caterpillar) for a shorter period of 12 h and the two larger species (grasshopper and roach) a period of 24 h.

### Breath sampling and tissue analyses

During starvation, all animals had access to hydrated gelatin polymer water crystals to maintain humidity within the metabolic chambers and provide them with drinking water so that starvation stress was not confounded with dehydration stress [45]. When hydrated this material is >99.9% water and does not provide a significant energy source during starvation. The metabolic chambers were ported to allow fresh air to passively circulate, but these ports were closed prior to gas sampling to ensure CO_2_ concentrations were between 2 to 4% at the time of breath collection. Gas samples were collected using gas tight syringes and injected into evacuated 12ml Exetainer^TM^ vials (Labco Limited; Ceredigion, UK). The metabolic cages were cleaned every other day to minimize microbial growth. The starvation trials were stopped once half of the experimental animals succumbed to starvation (i.e., lethal time; LT_50_). Because the LT_50_ includes total time food was withheld, it includes the period during which the insects were becoming postabsorptive and thus overestimates the actual ‘starvation’ period by 12 (cricket, and beetle and moth larvae) or 24 (grasshopper, cockroach) hours as described above.

The δ^13^C in each gas sample was analyzed using a HeliFAN Plus nondispersive infrared spectrometer (Fischer, ANalysen Instrumente GmbH; Germany) interfaced with a FANas autosampler. The ^13^C-analyzer was internally and externally calibrated at the start of each batch of samples. Vials containing a standard gas (2.5% CO_2_; Mesa Specialty Gases) were analyzed after every five unknown breath samples to identify and correct for analytical drift.

Postabsorptive insects were selected from each population and killed at the start of the starvation trials to measure their initial tissue δ^13^C. The carcasses were dried to a constant mass at 70°C in a convection oven, ground with a mortar and pestle, and further homogenized using a dental amalgamator. The lipid and lean fractions of the carcasses were chemically isolated using a modified Folch extraction method [46]. The δ^13^C of each fraction was analyzed at the University of Arizona using a Picarro (Sunnyvale, CA) G2121-i Cavity Ring Down Spectroscopy (CRDS) δ^13^C stable isotope analyzer with the A0502 ambient CO_2_ interface, an A0201 Combustion Module, and an A0301gas interface (CM-CRDS). All ^13^C concentrations are expressed in δ^13^C_VPDB_ [47,48].

### Calculations and statistics

Because the amount of ^13^C in the exhaled breath is primarily a function of the absolute concentration of the ^13^C in the diets, reporting of the actual δ^13^C of the breath provides only limited insight [49]. It is more informative to document how the δ^13^C of the breath changes as the insects gradually transition from the nourished state to starvation. We therefore used the δ^13^C from the first, postabsorptive breath samples as a starting reference point for each species. All of the subsequent δ^13^C breath values during starvation are expressed in terms of that reference point. For example, positive values mean that the breath of the starving animal contained a higher concentration of ^13^C than the recently postabsorptive animals, whereas negative values mean that the breath contained a lower concentration of ^13^C. We used a one sample t-test against a mean (SigmaPlot 12, San Jose, CA) at each time point to determine whether the δ^13^C in the breath significantly differed from the prestarvation values. Evaluations were made using Because we were asking whether a specific time point along a specific sequence was different from the initial value (and not more generally which values were different) there was no need for a Bonferroni-type correction and therefore α = 0.05.

^13^C-breath testing relies on the assumption that the oxidation and subsequent excretion of ^13^CO_2_ from the ^13^C-palmitic acid and ^13^C-leucine tracers is proportional to the rates of lipid and protein oxidation in the whole body. This assumption is not unrealistic because these two monomers comprise such a large component of their parent nutrient pools. For example, palmitic acid accounts for approximately 30% (21.8–46.5%) of the fatty acids in insects used for human consumption [50] and leucine accounts for approximately 7% (1–9%) of all of the amino acid residues in body proteins [50]. It is worth noting that studies of vertebrates have shown that the relative amount of leucine, an essential amino acid, in the body proteins remained constant despite substantial protein losses over a six-month starvation period [51] supporting our contention that leucine content is an accurate proxy for total protein levels.

The δ^13^C of the exhaled breath of starving animals, either reported in absolute terms or in terms of the difference between the freshly postabsorptive and the starved states as described above, follows highly complex time-dependent functions. The complexities of these functions are not unlike those seen during the *specific dynamic action* (SDA) response in postprandial animals. We therefore borrowed and modified some of the descriptive variables routinely used to characterize SDA responses among animals (see [52,53]) to describe the changes in lipid and protein oxidation during starvation. Specifically, we defined several metrics including:

- LT_50_:- The time (in days) after which food was removed required for 50% of the individuals of a species to succumb to starvation.
- lipid_peak_:- The time (in days) at which peak lipid oxidation occurred denoted by the maximal δ^13^C in the breath of the palmitic acid treatment groups.
- lipid_peak_% LT_50_:- The relative timing of the lipid_peak_ expressed in terms of a percent of LT_50_.
- protein_minimum_:- The time (in days) at which minimum protein oxidation occurred denoted by the minimum δ^13^C in the breath of the leucine treatment groups.
- protein_minimum_% LT_50_:-The relative timing of the protein_minimum_ expressed in terms of a percent of LT_50_.
- sparing_duration_:- The time (in days) during which protein sparing was occurring. Protein sparing was defined as the period during which the mean δ^13^C in the breath of the leucine treatment was lower (negative) during starvation than in freshly postabsorptive insects.
- sparing_duration_ % LT_50_:- The relative length of sparing_duration_ expressed in terms of a percent of LT_50_.
- protein_peak_:- The time (in days) at which peak protein oxidation occurred denoted by the maximal δ^13^C in the breath of the leucine treatment groups.
- protein_peak_ % LT_50_:- The relative timing of protein_peak_ expressed in terms of a percent of LT_50_.

### Strategies for maximal longevity

We addressed the question of what the best strategies of resource utilization are to maximize longevity, by plotting the relationships between LT_50_ *versus* lipid_peak_, protein_peak_, and sparing_duration_. We fit either a linear or a non-linear exponential regression whichever had the higher coefficient of determination (R^2^).

## Results

### Tissue enrichment

The tissues of the growing insects became enriched in ^13^C roughly in the proportions that their respective diets were isotopically enriched (Table 2). For example, the highest δ^13^C values were seen in tissues of the moth larvae raised on 8g kg^-1^ (dry mass) of tracers in their prepared diet and the lowest enrichments were seen in the tissues of the beetle larvae raised on unlabeled bran supplemented with tilapia food spiked with 1g kg^-1^ of ^13^C-tracer. In general the ^13^C from the leucine tracer remained in the nonlipid pool and the ^13^C from the palmitic acid tracer remained in the lipid pool (Table 2). See the Discussion for more details.

**Table 2:** Isotopic enrichment of the lean and lipid fractions in the whole bodies of insects raised on diets supplemented with either ^13^C-leucine or ^13^C-palmitic acid. See Table 1 for dosing levels.

Species	Treatment	Fraction	δ^13^C	s.d.
*G*. *portentosa*	palmitic acid	lipid	22.4	3.5
	palmitic acid	lean	-12.8	0.9
	leucine	lipid	-21.5	0.2
	leucine	lean	3.5	4.3
*A*. *domesticus*	palmitic acid	lipid	33.7	1.6
	palmitic acid	lean	-13.0	1.2
	leucine	lipid	-22.5	0.9
	leucine	lean	29.7	4.1
*R*. *microptera*	palmitic acid	lipid	39.3	2.9
	palmitic acid	lean	-12.1	1.6
	leucine	lipid	-16.8	1.6
	leucine	lean	43.4	3.4
*Z*. *morio*	palmitic acid	lipid	33.0	2.7
	palmitic acid	lean	-13.0	1.3
	leucine	lipid	-18.1	0.9
	leucine	lean	52.1	3.8
*M*. *sexta*	palmitic acid	lipid	176.6	72.3
	palmitic acid	lean	5.9	8.5
	leucine	lipid	-2.7	3.3
	leucine	lean	123.3	61.7

### Oxidation of endogenous lipids

**Fig 1:**
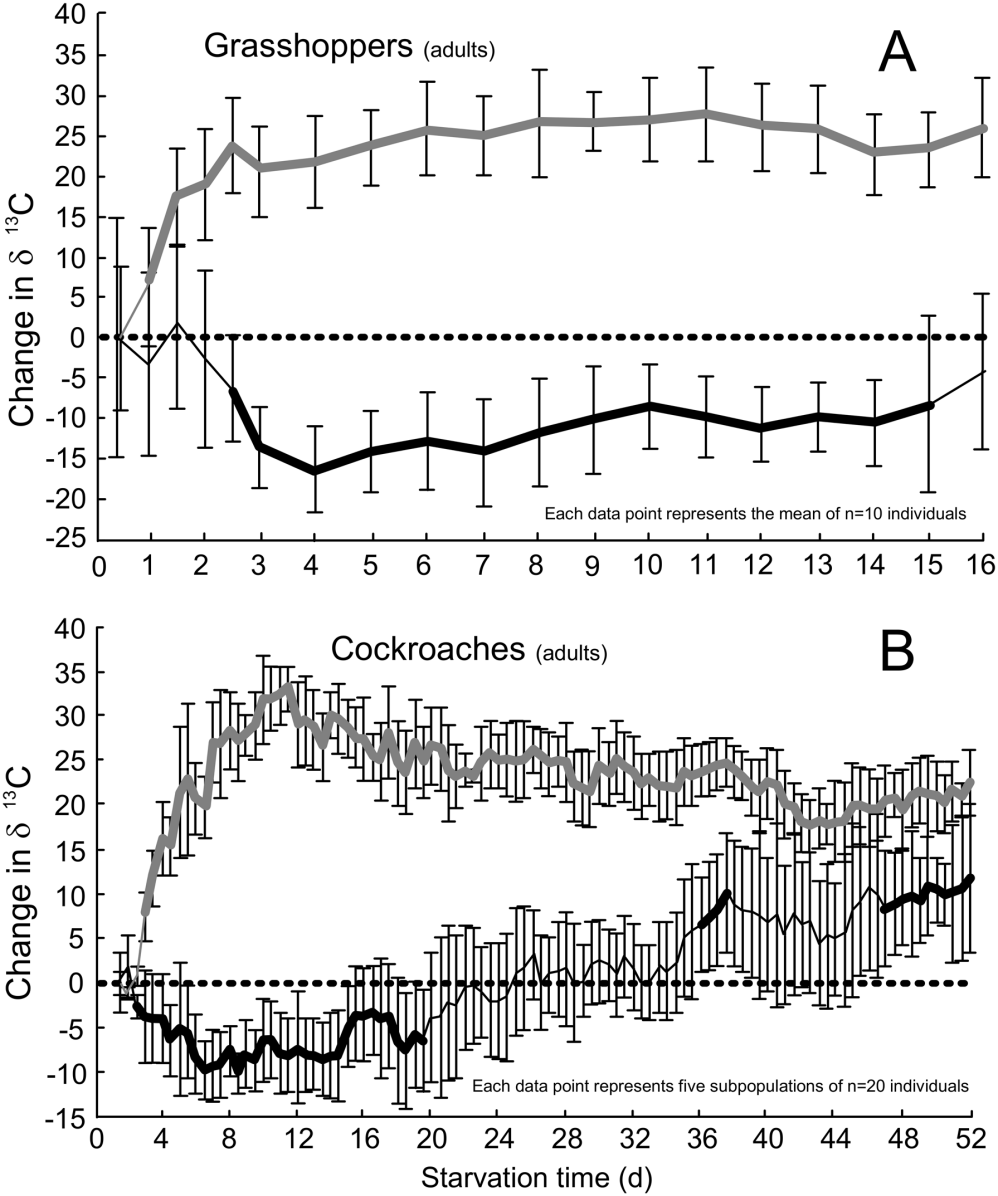
Starvation-induced changes in the δ^13^C of the exhaled breath of insects. Adult grasshoppers (A) and cockroaches (B) raised on diets supplemented with ^13^C-palmitic acid tracers (grey) or ^13^C-leucine tracers (black). The dashed line represents the δ^13^C of the breath in postabsorptive insects at the start of fasting. Error bars represent standard deviations. The bold lines indicate time points at which fasting values were statistically different from the prefasting values according to two-way, one-sample t-tests.

**Fig 2:**
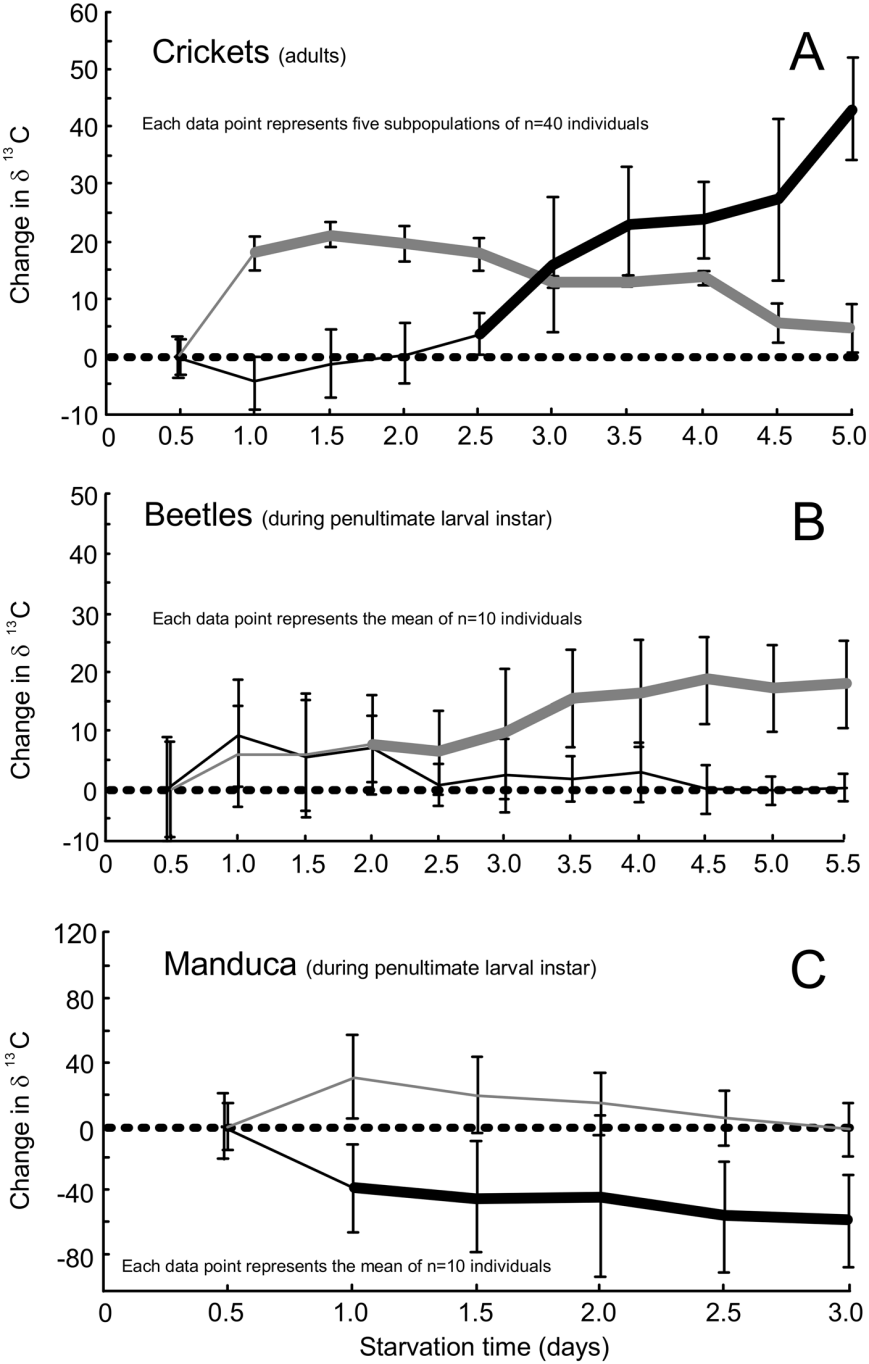
Starvation-induced changes in the δ^13^C of the exhaled breath of insects. Adult crickets (A) and larval beetles (B) and moths (C) raised on diets supplemented with ^13^C-palmitic acid tracers (grey) or ^13^C-leucine tracers (black). The dashed line represents the δ^13^C of the breath in postabsorptive insects at the start of fasting. Error bars represent standard deviations. The bold lines indicate time points at which fasting values were statistically different from the prefasting values according to two-way, one-sample t-tests.

All of the insects increased their rate of lipid oxidation at the onset of starvation, but the responses thereafter were species-specific. The starving grasshoppers quickly increased lipid oxidation and maintained a high reliance on lipid oxidation that peaked at 11 days (lipid_peak_), 69% of the LT_50_ (Table 3). Immediately preceding death their reliance on lipids remained significantly higher than prestarvation values (Fig 1A). The cockroaches, crickets, and larval moths exhibited their lipid_peak_ during the first third of the starvation period (Table 3), but near the point at which they succumbed to starvation their reliance on lipids was varied. Immediately preceding death the cockroaches exhibited rates of lipid oxidation that were significantly higher than prestarvation levels (Fig 1B). In contrast, after reaching peak lipid oxidation early in the starvation period, the moth larvae gradually reduced their reliance on lipid oxidation (Fig 2C). In the moth larvae these values were not significantly different form the prestarvation values (Fig 2A). The beetle larvae were unique in that they did not rapidly increase lipid oxidation at the onset of starvation and only reached peak lipid oxidation (lipid_peak_) near the point of death (Fig 2B). At no time point did any of the starving insects exhibit rates of lipid oxidation that were significantly lower than the prestarvation states; however, this was not the case with protein oxidation (see below).

**Table 3:** Measures of starvation performance and lipid and protein oxidation. See Methods for details about calculations.

	LT_50_	Lipid_peak_	Lipid_peak_	Protein_minimum_	Protein_minimum_	Sparing_duration_	Sparing_duration_	Protein_peak_	Protein_peak_
Species	(days)	(days)	(%LD_50_)	(days)	(%LD_50_)	(days)	(%LD_50_)	(days)	(%LD_50_)
*G*. *portentosa*	52	11.5	22.1	8.5	16.3	21	40.4	52	100.0
*A*. *domesticus*	5	1.5	30.0	1	20.0	1.5	30.0	5	100.0
*R*. *microptera*	16	11	68.8	4	25.0	14	87.5	16	100.0
*Z*. *morio*	5.5	4.5	81.8	5	90.9	0	0.0	1	18.2
*M*. *sexta*	3	1	33.3	3	100.0	2.5	83.3	0.5	16.7

### Oxidation of endogenous proteins

The responses with regard to protein oxidation were more complex than those described above for lipid oxidation. Four of the five insect species exhibited some tendency for protein sparing whereby their reliance on protein oxidation was lower than it was in the prestarvation state, but this response was only significant in the grasshoppers, cockroaches, and moth larvae. Protein_minimum_ occurred during the first quarter of the experiment in cockroaches, crickets, and grasshoppers. These three species were apparently able to spare proteins for 30% to 88% of the starvation period (sparing_duration_; Table 3). Although the moth larvae were also able to spare proteins for most of the experiment, they exhibited minimum rates of protein oxidation only at the end of the experiment (Fig 2C). The beetle larvae were apparently unable to reduce protein oxidation below prestarvation values at any time point (Fig 2B). Notably, the beetle larvae exhibited peak protein oxidation 18% into the experiment (protein_peak_; Table 2)–a point at which most other species were exhibiting protein sparing.

At the time of death the reliance on protein oxidation was as varied among the five species as it was for lipid oxidation. For example, the cockroaches and crickets were oxidizing proteins at or near their peak rates that were also significantly higher than prestarvation rates. In contrast, the grasshoppers and beetle larvae relied on protein oxidation at the time of death to an extent similar to their prestarvation levels. The moth larvae were unique in that they exhibited their minimal reliance on protein oxidation immediately preceding death at rates that were significantly lower than prestarvation rates.

### Strategies of resource use

**Fig 3:**
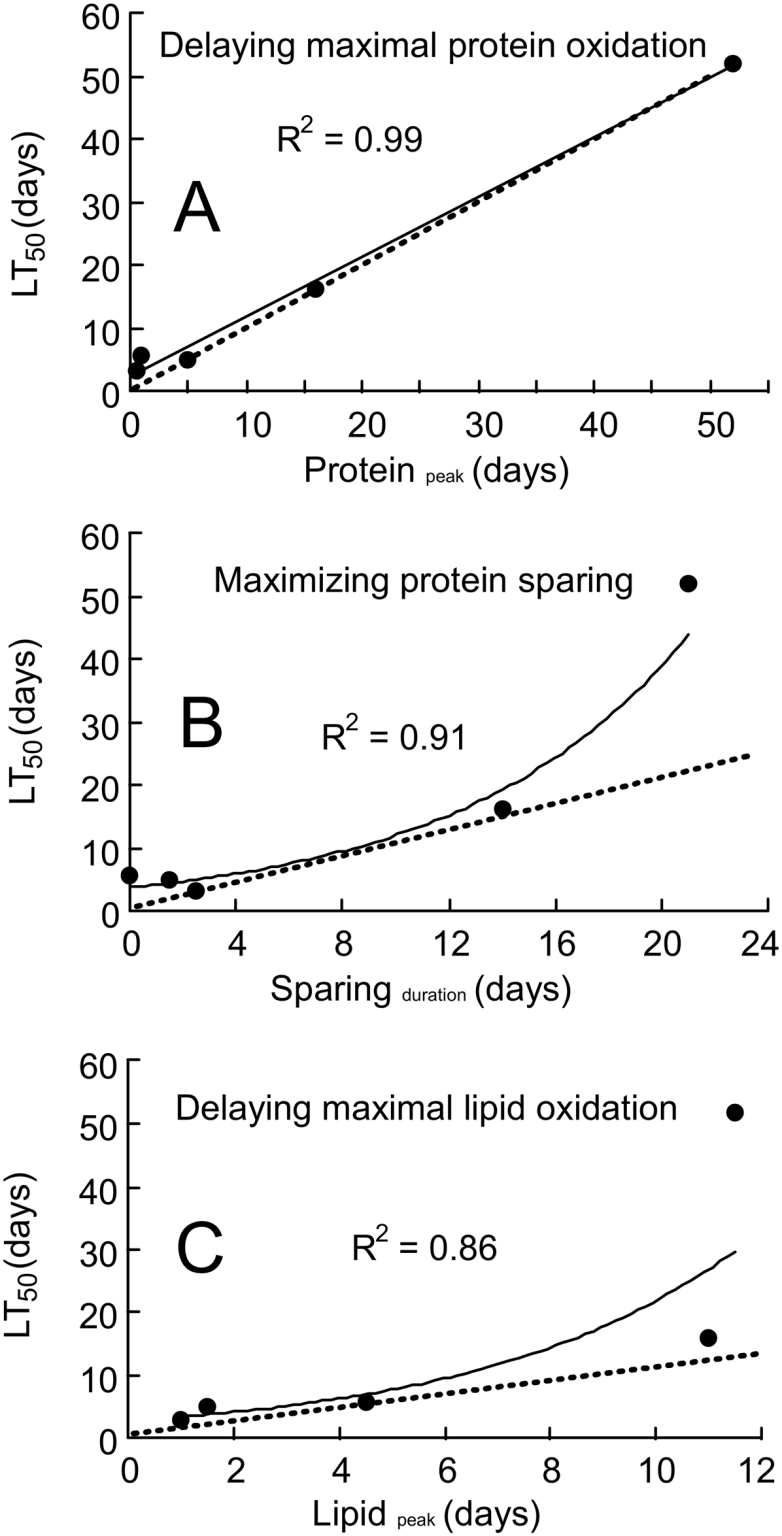
Correlations between fuel use and longevity. Relationships between starvation tolerance (LT_50_) and physiological metrics related to fuel oxidation in five species of insects (data from Table 2). The dashed lines illustrate isometry.

Protein_peak_ showed a linear relationship with LT_50_ (y = 0.9475x + 2.1826, R^2^ = 0.99, Fig 3A). Lipid_peak_ and sparing_duration_ showed non-linear relationships with LT_50_ (sparing_duration_: y = 3.7105e^0.1175x^, R^2^ = 0.91, Fig 3B; lipid_peak_: y = 2.7102e^0.2085x^, R^2^ = 0.86, Fig 3C). There were no significant relationships between LT_50_ and any of the other metrics defined above suggesting these were not as important in determining starvation tolerance.

## Discussion

### ^13^C Tracer integration into the body

The ^13^C-leucine and ^13^C-palmitic acid tracers added to the insect diets were effective at enriching the lean and lipid fractions in the bodies, respectively (Table 2). Experimentally controlling the dose of ^13^C-tracers was more effective for species that consumed a homogenized, prepared diet (Table 1) than those that specialized on bran or fresh lettuce (mealworms and grasshoppers, respectively), however it may be possible to individually force feed a fixed amount of tracer to larger species (e.g., grasshoppers; J.D. Hatle, unpublished observation).

Interestingly, in most cases the δ^13^C of the body lipids of the palmitic acid groups was higher than the δ^13^C of the lean tissue of the conspecific leucine groups (Table 2). The higher tissue ^13^C enrichment within the lipids was initially surprising given that the molecular mass of palmitic acid is nearly twice that of leucine and thus the insects in the leucine treatments ultimately consumed nearly twice the number of tracer-derived ^13^C-atoms than those in the palmitic acid treatment. We did not quantify the rates of exogenous (i.e., dietary) tracer oxidation in the insects during growth, but previous studies on birds [54,55], rodents [56], and bats [57], have shown that exogenous leucine is oxidized at a rate nearly an order of magnitude greater than palmitic acid during the postprandial phase. Comparisons of the oxidation of ^13^C-labeled leucine and oleic acid (another common fatty acid) in postprandial grasshoppers shows that 1.6% and 0.64% of the exogenous ^13^C was recovered, respectively, in the breath by 11 hours [58]. Previous studies of crickets injected with ^14^C-radiolabeled tracers into their hemocoel showed that the amino acid, glycine was oxidized about three-fold more rapidly than palmitic acid [59,60]. Consequently, the observed differences in δ^13^C between the lipid and lean pools in the nourished insects may be explained by the fact that less exogenous palmitic acid was oxidized thereby leaving more to be allocated to the growing tissues [49].

A negligible amount of the ^13^C from the leucine tracer was bioconverted into the lipid pool. The mean δ^13^C of the lipids in control populations (populations that were not exposed to any ^13^C-tracers) of cockroaches and crickets (-22.4‰) was not statistically different from the mean δ^13^C of the lipids in cockroaches and crickets in the ^13^C-leucine treatment (-22.0‰; t-test, df = 14, p = 0.107). We did not raise parallel, control populations of the other species, but we assume the basic biochemistry of these insects is generally similar. The minimal ‘leakage’ (*sensu* [35]) of the ^13^C from the leucine tracer was similar to that reported for mice raised for seven weeks on ^13^C-leucine tracers.

A small amount of the ^13^C from the palmitic acid tracer was recovered in the lean tissues of the insects. The mean δ^13^C of the lean tissue in control populations of cockroaches and crickets (-18.5‰) was statistically different from the mean δ^13^C of the lean tissue in cockroaches and crickets in the ^13^C-palmitic acid treatment (-12.9‰; t-test, df = 14, p < 0.001). This extent of ^13^C leakage from the lipid pool into the lean pool was greater than previously reported in mice raised on ^13^C-palmitic acid [35], yet it remains unclear precisely how the ^13^C from the palmitic acid tracer was partitioned between the carbohydrate and protein components of the lean tissue fraction because we did not analyze these fractions separately. Compound specific stable isotope analyses would be useful to determine how the palmitic acid-derived ^13^C atoms become distributed among non-lipid components [61–63]. Nevertheless, we estimate that >95% of the ^13^C atoms from the palmitic acid tracer remained within the lipid pool of the body, and conclude that the δ^13^C in the exhaled breath of the palmitic acid treatment groups was an effective proxy of endogenous lipid oxidation during starvation.

### What strategies contribute to improved starvation tolerance?

The LT_50_ ranged from 3 days in moth larvae to 52 days in adult cockroaches. Comparisons of starvation tolerance across studies are difficult given the dearth of values reported in literature for these species and life stages. American cockroaches apparently begin to succumb to starvation within two weeks [17,64], and all died by 42 days [65]. Many of the cockroaches in this study lived beyond 60 days. Studies of *Gryllus* crickets report that they are capable of surviving starvation for two days longer than LT_50_ of the *Acheta* species in the present study [15,26]. While we did observe a few crickets tolerating at least seven days of starvation we do not consider these to be representative of the general population.

Some crickets and cockroaches are known to reduce their locomotor activity during starvation [26,65]. The crickets and the cockroaches in this study were maintained in groups, and not individually, which may have increased activity and affected their apparent starvation tolerance. We eventually improved our sampling protocols so that we could measure the breath on individuals of the other three focal species. The three-day LT_50_ for the penultimate instar moth larvae is not surprising as other work has shown that these moths enter their last larval instar with almost no fat reserves (Helm and Davidowitz, unpublished data).

A superficial perspective reveals that starvation tolerance in this study was positively correlated with body mass, but we consider this to be a spurious correlation and an artifact of our selection of two large species and three species (or life stages) that were an order of magnitude smaller (see Methods). Positive correlations have been made between starvation tolerance and body mass in *Drosophila* [21], ant lion larvae [8], and backswimmers [14], but bumble bees show the opposite relationship [66], and we have no evidence that body size in insects is optimized to improve starvation tolerance as it may in mammals [67,68]. Measures of starvation tolerance among other insect species will be useful to confirm this.

Researchers recently developed a dynamic energy budget model to describe different physiological responses to starvation in insects [14]. The model includes three strategies that insects might employ including 1) reducing somatic maintenance costs, 2) maximizing the mobilization of endogenous nutrients, and 3) regulating energy mobilization to only pay for maintenance costs. It was not an objective of this study to document the extent to which these insects engaged in starvation-induced hypometabolism as has been done for many vertebrate species (reviewed in [3,69]). One study on starving *Gryllus* crickets found that they did not reduce metabolic rates during prolonged starvation [15], but future studies designed to document changes in standard metabolic rates and activity levels will be useful to document energy saving strategies during starvation in other species. Nevertheless our results do provide insight into the latter two, mutually exclusive, starvation strategies. If the starving insects were maximizing the rates of nutrient oxidation, we would expect to see evidence of increased protein and increased lipid oxidation at the onset of starvation. While the larval beetles exhibited a response weakly resembling this strategy, all of the other species exhibited inverse changes in endogenous protein and lipid oxidation that are suggestive of physiological regulation and not a state where all possible fuels were maximally oxidized.

The insects in this study exhibited varying strategies with regard to the regulation of fuel oxidation. Are these differences related to their ability to withstand starvation? We found several correlations between survival time (LT_50_) and particular performance metrics (Fig 3A). The strongest of these was a linear relationship between LT_50_ and protein_peak_. The relationship between LT_50_ and protein_peak_ was isometric supporting the long-standing idea that starvation tolerance is generally limited by an unregulated increase in protein oxidation. But the strategies occurring during the earlier phases of starvation might play a role in delaying this reliance on protein. If we assume that the five species used in this study are representative of insects in general (an assumption that needs to be tested in future studies), then correlations between the starvation tolerance and duration of protein sparing and the timing of maximal lipid oxidation (lipid_peak_) could offer insight into variation in starvation tolerance. In particular, all of the species besides the cockroaches exhibited roughly isometric relationships between sparing_duration_ and lipid_peak_ *versus* LT_50_ (Fig 3B and 3C). Interestingly, the values for the cockroaches did not follow these linear relationships, suggesting possible mechanisms responsible for the comparatively high starvation tolerance in the cockroaches. While each of the aforementioned relationships are correlative, they provide a basis for formulating hypotheses and predictions about which particular physiological strategies are responsible for differences in the starvation tolerance among insects. Future comparisons among the starvation strategies of other insects that exhibit *high* and *low* tolerances to starvation could be useful to examine this possibility. It would also be informative to correlate the timing of these physiological events with variation in starvation tolerance within conspecifics at the same life stage that may differ in their adiposity (*sensu* [70,71]); unfortunately, this would involve destructive sampling that would not be compatible with breath testing.

Starvation strategies of insects, like other life history traits, appear to be shaped in part by the life stage [60]. The adult cockroaches, grasshoppers, and crickets tended to exhibit inverse patterns of oxidation of their lipids and proteins. These species are income breeders and would normally allocate ingested food among the demands associated with maintenance, locomotion, and reproductive activity. However, many other insect species are capital breeders and may normally fast (*sensu* [72]) during adulthood [73]. It would be useful to characterize how those species partition their lipids and proteins to meet energy demands during adulthood. Growth is an imperative component of the life history of larval insects and the two larval species examined in this study exhibited comparatively low reliance on protein oxidation during starvation, suggesting that larval insects maximize protein sparing to support larval growth. Future studies comparing the starvation strategies among the different life stages of both holometabolous and hemimetabolous species will be useful to characterize how starvation strategies may change ontogenetically.

### Conclusion

The findings of this study underscore the growing awareness among comparative physiologists that starving animals do not necessarily follow the classic paradigm that canalizes the physiological progression into three discrete phases [74,75]. In fact, only two of the five species exhibited any evidence of sharply increased rates of protein oxidation immediately preceding death as predicted by Lusk (1928) and since retained as part of the paradigm in nutritional physiology [39]. Although there is a need for additional comparative studies of starvation physiology among many key groups of vertebrates and insects [76,77], the diverse responses we describe here raise the possibility that insects employ a broader range of strategies for regulating lipid and protein use than expected. Furthermore, the ability to regulate critical transitions in lipid and protein mobilization may help explain the differences in starvation tolerance, especially the physiological responses that immediately precede death. In addition to characterizing starvation responses in other insect species it will be important to explore the evolutionary underpinnings of this remarkable physiological diversity in the context of phylogenetic relationships and ecological factors.
